# Clinical–Epidemiological Characteristics and *IFITM-3* (rs12252) Variant Involvement in HIV-1 Mother-to-Children Transmission Susceptibility in a Brazilian Population

**DOI:** 10.3390/life13020397

**Published:** 2023-01-31

**Authors:** Dalila Bernardes Leandro, Ronaldo Celerino da Silva, Jessyca Kalynne Farias Rodrigues, Maria Carollayne Gonçalves Leite, Luiz Claudio Arraes, Antonio Victor Campos Coelho, Sergio Crovella, Luisa Zupin, Rafael Lima Guimarães

**Affiliations:** 1 Department of Genetics, Federal University of Pernambuco (UFPE), Avenida da Engenharia, S/N, Cidade Universitária, Recife 50670-901, PE, Brazil; 2 Keizo Asami Institute (iLIKA), Federal University of Pernambuco (UFPE), Avenida Prof. Moraes Rego, S/N, Cidade Universitária, Recife 50670-901, PE, Brazil; 3 Departament of Virology and Experimental Therapy (LAVITE), Aggeu Magalhães Institute (IAM), Oswaldo Cruz Foundation (Fiocruz), Avenida Prof. Moraes Rego, S/N, Cidade Universitária, Recife 50670-901, PE, Brazil; 4 Institute of Medicine Integral of Pernambuco Professor Fernando Figueira (IMIP-PE), Rua dos Coelhos, 300, Boa Vista, Recife 50070-902, PE, Brazil; 5 Hospital Israelita Albert Einstein, Av. Albert Einstein, 627/701-Morumbi, São Paulo 05652-900, SP, Brazil; 6 Biological Science Program, Department of Biological and Environmental Sciences, College of Arts and Sciences, Qatar University, Doha 2713, Qatar; 7 Institute for Maternal and Child Health—IRCCS “Burlo Garofolo”, 34137 Trieste, Italy

**Keywords:** HIV-1, *IFITM-3*, mother-to-children transmission, viral restriction factor, clinical epidemiological factors

## Abstract

**Simple Summary:**

Mother-to-children transmission (MTCT) is the main infection route of HIV-1, mainly occurring in pregnancy, delivery, and/or postpartum and it is a multifactorial phenomenon, where genetic variants play an important role. A case–control study was performed in HIV-1 infected mothers and their exposed infected and uninfected children from Pernambuco, Brazil. Our analysis shows that transmitter mothers have a significantly lower age at delivery, late diagnosis, deficiency in ART use (pregnancy and delivery), and detectable viral load in the third trimester of pregnancy compared with non-transmitter mothers. Infected children show late diagnosis, vaginal delivery frequency, and tend to breastfeed, differing significantly from uninfected children. Moreover, the genetic analysis reveals that a variant in the *IFITM-3* gene (an important viral restriction factor) is significantly more frequent among infected than uninfected children.

**Abstract:**

Mother-to-children transmission (MTCT) is the main infection route for HIV-1 in children, and may occur during pregnancy, delivery, and/or postpartum. It is a multifactorial phenomenon, where genetic variants play an important role. This study aims at analyzing the influence of clinical epidemiological characteristics and a variant (rs12252) in interferon-induced transmembrane protein 3 (*IFITM-3*), a gene encoding an important viral restriction factor, on the susceptibility to HIV-1 mother-to-children transmission (MTCT). A case–control study was performed on 209 HIV-1-infected mothers and their exposed infected (87) and uninfected (122) children from Pernambuco, Brazil. Clinical–epidemiological characteristics are significantly associated with MTCT susceptibility. Transmitter mothers have a significantly lower age at delivery, late diagnosis, deficiency in ART use (pregnancy and delivery), and detectable viral load in the third trimester of pregnancy compared with non-transmitter mothers. Infected children show late diagnosis, vaginal delivery frequency, and tend to breastfeed, differing significantly from uninfected children. The *IFITM-3* rs12252-C allele and TC/CC genotypes (dominant model) are significantly more frequent among infected than uninfected children, but the statistical significance does not remain when adjusted for clinical factors. No significant differences are observed between transmitter and non-transmitter mothers in relation to the *IFITM-3* variant.

## 1. Introduction

Mother-to-children transmission (MTCT) is the main infection route for HIV-1 in children under 13 years, occurring in pregnancy and delivery, or postpartum through breastfeeding. In 2019, it was responsible for about 1.8 million children living with HIV-1 around the world [1].

Even in the absence of preventive measures, 55% to 85% of exposed children will not be infected [2], revealing that MTCT has a complex and multifactorial nature. Several clinical–epidemiological characteristics, such as antiretroviral therapy (ART) use, viral load, and CD4+ T cell levels, may contribute to MTCT. Viral and host genetic factors can also be related to HIV-1 MTCT susceptibility, such as viral restriction factors gene variants [3,4,5,6]. 

Interferon-induced transmembrane protein 3 (IFITM-3), encoded by *IFITM-3* in chromosome 11 (11p.15.5), is a viral restriction factor, constitutively expressed in various tissues (barrier epithelial cells, oral mucosa, esophagus, and placenta), mainly by the immune system cells (macrophages and T lymphocytes) [7,8].

IFITM-3 inhibits many enveloped viruses, including HIV-1 [9,10,11,12,13], by alteration of biophysical properties [14,15] and the cholesterol content of host cell membranes [16], making them more refractory against viruses’ entry [17]. These proteins are also present in the internal membranes of the endoplasmic reticulum, endosomes, and lysosomes, regulated by different post-translational modifications [11,17,18,19,20], which can modulate their antiviral function [18,19].

Genetic variants in human *IFITM-3* have been related to the modulation of gene function [21,22,23,24,25]. The rs12252 C allele can alter a splice acceptor site, resulting in a truncated protein lacking the N-terminal 21 amino acids (IFITM-3∆21) [26]. The main binding motifs, responsible for IFITM-3 location in the cell, are in the N-terminal region; therefore, during biosynthesis IFITM-3∆21 protein is trafficked to the plasma membrane, but not endocytosed, leading to its accumulation [18] and allowing a physical blockage of viral entry [23,24,25,26].

In the context of HIV-1 infection, in vitro studies suggested that IFITM-3∆21 was associated with lower susceptibility to infection, by decreasing the infectivity of nascent viral particles, cell–virus fusion [27,28], and replication inhibition [11]. However, how *IFITM-3* variants may influence HIV-1 infection susceptibility is still an open question. Indeed, it was suggested that the rs12252 C allele was associated with rapid disease progression, but not HIV-1 infection in a Chinese cohort [21].

Considering the IFITM-3 antiviral function and its expression in tissues such as placenta and oral mucosa, the present study analyzed the influence of *IFITM-3* rs12252 polymorphism and clinical–epidemiological variables on HIV-1 MTCT susceptibility in a northeastern Brazilian population.

## 2. Material and Methods

### 2.1. Study Design

A retrospective case–control study was performed with 209 HIV-1 infected mothers and their respective exposed children (87 infected and 122 uninfected). Mothers/children were recruited at the Institute of Integral Medicine of Pernambuco Professor Fernando Figueira (IMIP-PE) in Recife (northeast Brazil), from 2013 to 2016.

Inclusion criteria were HIV-1 infected mothers who had at least one detectable viral load (VL) (>40 copies/mL) during pregnancy, antiretroviral therapy (ART) use, and their respective children exposed to HIV-1 via MTCT under 13 years of age. The exclusion criteria were individuals without complete clinical parameters in medical records; mothers and their respective children who maintained VL undetectable throughout the gestational period; children infected by other transmission routes than MTCT. All individuals enrolled in the study were from the same geographical origin, the metropolitan area of Recife, Pernambuco, Brazil.

All individuals involved in the research had their medical records reviewed. The following information was collected from the children’s medical records: sex, age at diagnosis, viral load, birth weight, type of delivery, and breastfeeding history. From the mothers, data were collected regarding childbirth, maternal diagnosis, use of ART (pregnancy and childbirth), and viral load during pregnancy.

The mothers were categorized as transmitters (case) and non-transmitters (controls). The exposed children were categorized as infected (at least two detectable VL, and/or positive HIV-1 serology) and uninfected (minimum of two undetectable VL, and/or negative HIV-1 serology). The exposed infected children were considered as the case group, while the exposed uninfected children were considered as controls.

All methodological procedures were assessed and approved by the IMIP-PE Human Research Ethics Committee (nº 2629-13).

### 2.2. DNA Extraction and Genotyping

Mothers’ and children’s genomic DNA was extracted from peripheral blood, using mini salting-out protocol [29].

The *IFITM-3* rs12252 SNP was genotyped using allelic specific probes (TaqMan^®^ SNP Genotyping Assays C_175677529_10) in ABI7500 Real-Time PCR (Applied Biosystems, Thermo Fisher Scientific, Waltham, MA, USA), according to manufacturer’s recommendations.

Additionally, aiming to minimize possible confounding genetic factors well-known to be associated with susceptibility to HIV-1 infection, the *CCR5*Δ32–rs333 (genetic factor associated with HIV-1 entry susceptibility protection) genotyping was performed by polymerase chain reaction (PCR), using the primer sequences: forward 5′-GTCTTCATTACACCTGCAGCTCT-3′ and reverse 5′- CACAGCCCTGTGCCTCTT-3′. The amplicons were analyzed by 3% agarose gel electrophoresis using ethidium bromide as staining.

### 2.3. Statistical Analysis

Allelic and genotypic frequencies were estimated by direct counting using genotype transposer [30]. Hardy–Weinberg equilibrium (HWE) adherence and possible associations were verified through chi-square test (X^2^) and Fisher’s exact test, respectively.

Shapiro–Wilk test was used to assess if specific variables distribution agreed to a normal distribution before univariate statistical analyses; if they did not, Mann–Whitney tests were used for quantitative variables. Categorical variables were compared by Fisher’s exact test. All tests were two-tailed with a significance level of α = 0.05 and performed using R program version 2.11.1 [31] or GraphPad Prism version 5.0 (GraphPad Software, La Jolla, CA, USA).

Additionally, we performed a multivariable logistic regression to assess any genetic association while controlling for all clinical–epidemiological data, also using R program. We included interaction terms to assess if there were any discernible effects of mother– child genotype interaction leading to susceptibility to or protection against MTCT.

The statistical power analysis of the study was performed using the G*power 3.1.9.7 software.

## 3. Results

The clinical–epidemiological characteristics of HIV-1 infected mothers and their respective children are displayed in Table 1.

Transmitter mothers show a lower median age at delivery (23.3 years), differing significantly from non-transmitter mothers (26.1 years, *p* = 0.011). Regarding the diagnosis, previous knowledge of HIV-1 serological status (before prenatal care) is significantly more frequent among non-transmitter mothers (41.8%) than transmitter mothers (4.6%; *p* = 0.009). The delayed acknowledgment (HIV-1 diagnostic at delivery and postpartum) is more common among transmitter mothers (21.8% and 51.7%, respectively) than in non-transmitter mothers (11.5%, *p* = 0.002; and 0.8%, *p* = 2.2e^−16^, respectively).

In relation to antiretroviral therapy, non-use during gestation and delivery is significantly predominant among transmitter mothers (89.6% and 69%, respectively) in comparison with non-transmitter mothers (23.8%, *p* = 2.2e^−16^ and 25.4%, *p* = 8.7e^−12^, respectively), as expected.

The detectable VL in the third trimester of gestation is significantly more frequent in transmitter mothers (96.5%) than in non-transmitter mothers (66.4%; *p* = 7.4e^−8^). Significant differences are not observed for other pregnancy periods.

In relation to the children, most are female (>54.0%) with HIV-1 negative serology (58.4%) and with weight considered as normal (>48.0%). Univariate analysis shows a higher median of diagnosis age in infected children (0.96 years) than uninfected children (0.12 years, *p* = 0.0001). In addition, the frequency of uninfected children with insufficient weight (36.1%) is significantly higher than among infected children (13.8%, *p* = 0.0004).

Regarding the delivery, infected children are born mostly through vaginal delivery (55.2%), differing significantly from uninfected children (28.7%, *p* = 5.1e^−05^). Similarly, most infected children are breastfed in HIV-1+ mother (54%), differing significantly from uninfected children (3.3%, *p* = 2.2e^−16^).

Allelic and genotype distribution of *CCR5* and *IFITM-3* polymorphisms are shown in Table 2.

The *CCR5* and *IFITM-3* genotype distribution in mothers and children are in accordance with HWE.

The rs12252-C allele is significantly more common in infected (20.2%) than uninfected children (11%, *p* = 0.014). Statistically significant differences are observed between infected and uninfected children by dominant model, with the TC/CC genotypes significantly more frequent among infected children (OR = 2.07, *p* = 0.034). Although no statistically significant differences are found in the codominant model, some trends are observed, resulting in levels of statistical significances closer to 0.05. Indeed, the TT genotype is the most frequent among infected (64.3%) and uninfected children (78.9%), and the TC and CC genotypes are more frequent in infected (30.9% and 4.8%, respectively) than uninfected children (20.2% and 0.9%, respectively). The analysis of *IFITM-3* rs12252 is corrected by the presence of CCR5∆32 and the described association remains. For *IFITM-3* SNP, the statistical power value is >0.99 among children exposed to HIV-1.

Additionally, we also performed an analysis of concordance and discordance of *CCR5∆32* and *IFITM-3* rs12252 genotypes between exposed children and their respective mothers; however, no significant differences are observed (Supplementary Table S1).

The multivariable logistic regression reveals that late diagnosis (especially diagnosis around the time of childbirth (OR = 4.46, 95%CI = 1.14–17.41, *p*-value = 0.03) and breastfeeding (OR = 27.52, 95%CI = 1.62–465.68, *p*-value = 0.02) is associated with higher risk of HIV MTCT. No other statistical associations are observed, including genetic associations. No mother–child genotype interactions are detected either (Table 3).

## 4. Discussion

HIV-1 MTCT susceptibility presents itself as a multifactorial and complex event, dependent of viral, maternal, and pediatric variables. In our study population, maternal characteristics such as low age at delivery, late diagnosis, no use of ART during pregnancy/delivery, and detectable VL in the last pregnancy trimester are associated with increased susceptibility to HIV-1 MTCT, corroborating with previous studies [32,33,34].

Younger mothers may lack experience to care for themselves and their infants, and possibly have a less favorable socioeconomic status than older women [31]. These facts can directly impact other preventive measures, leading to late diagnosis and inappropriate ART use, maximizing the transmission risks, as observed in our and other populations [33,34,35].

Undetectable VL maintenance in gestation proved to be of great importance for preventing viral transmission. The HIV-1 MTCT occurs mainly during the third trimester of gestation by reduction in the placental vascular integrity [2], as we observed in our group.

The lack of an early maternal diagnosis leads to a child’s late diagnosis. In our population, infected children were born mostly through vaginal delivery, were breastfed, and had a higher age of diagnosis, corroborating with reports in the literature [36,37,38,39,40]. During vaginal delivery, infants are exposed to HIV-1 via fluids from the birth canal that penetrate their oropharyngeal cavity [36]. The elective cesarean delivery is an efficacious intervention for HIV-1 MTCT risk mitigation among infected mothers not taking ART [37].

In our sample, non-exclusive breastfeeding is predominant among infected children, which may be related to failures in health services. Most recruited mothers live far from large city centers, and, therefore, have precarious access to health services, leading to lack of precocious diagnosis/treatment. Napyo et al. [38] suggested that HIV-infected women with delivery not supervised by healthcare teams and without ART adherence were less likely to avoid exclusive breastfeeding, corroborating, at least in part, our results. In our sample, most of the deliveries were supervised by health workers, but deficiencies during care prevented the adoption of appropriate measures.

The insufficient weight in uninfected children was observed as a factor for lower susceptibility to infection, disagreeing with a report in a Zambian population [41], which did not find association between this variable and HIV-1 MTCT susceptibility. This result could be related to ART use in pregnancy, not observed in Zambia [41]. Thus, the ART use before or in the beginning of first trimester of pregnancy may increase the probability of the child being born with a lower weight [42].

Additionally, the rs12252-C allele of *IFITM-3* is related to susceptibility to HIV-1 MTCT in our study group, differing from what is expected for this variant [11,22]. The presence of rs12252-C polymorphism in *IFITM-3* can alter a splice-accepting site, encoding a protein lacking 21 amino acid residues [26] modifying the IFITM3∆21 protein solely in host cell membrane, thus, meaning a greater expected viral restriction. However, it is not completely clear whether the mutated protein, although present on the cell surface, maintains its functionality in viral restriction. In fact, Jia et al. [11] observed in vitro that IFITM3∆21 was more efficient in restricting the HIV-1 entry into the cell, being more abundantly incorporated into nascent viral particles than its wild form [22]. Therefore, the genetic association observed in our study needs to be confirmed in further functional studies.

Zhang et al. [21], investigating a Chinese cohort, observed that the rs12252-C allele is associated with a faster progression to AIDS, but not to HIV-1 susceptibility. The differences between the studies may lie in the studied groups, since Zhang et al. [21] compared HIV-1 infected adults against healthy men who have sex with men (MSM), while in our study, we analyzed exposed infected and uninfected children. In addition, Zhang et al. also observed that individuals with CC/CT risk genotypes exhibited higher viremia peaks, lower CD4+ T cells counts, and a greater risk for the rapid decline in the CD4+ T cells counts to levels below 350 cells/mL, when compared to control individuals [21].

Compton et al. [27], comparing different proteins of IFITM family, suggested that the IFITM-1 protein, which naturally does not have the N-terminal portion seen in IFITM-3, resembling IFITM3∆21, has a moderate restriction of HIV-1 infectivity compared to IFITM-3. In this sense, it is possible that, due to a still unknown mechanism, just being present in the plasma membrane is not sufficient to restrict the complete HIV-1 entry, the N-terminal portion being necessary for a more effective restriction, which could partially explain our findings.

## 5. Conclusions

Our results show the utmost importance of clinical–epidemiological characteristics in the susceptibility of HIV-1 MTCT, reinforcing the crucial role of a satisfactory prenatal screening provided by the health care system to mitigate HIV-1 transmission risks. In addition, being aware of the limitations (sample size, absence of some clinical variables, and functional validation), our study describes, for the first time, the association of the rs12252-C variant in *IFITM-3* with susceptibility to HIV-1 MTCT, suggesting the importance of this gene in modulating susceptibility to viral entry.

## Figures and Tables

**Table 1:** Clinical and epidemiological characterization of children exposed to HIV-1 via vertical transmission and their respective mothers from a Pernambuco state population.

Variables	Children Exposed to HIV-1		Non-Parametric and Univariate Tests OR (95%CI), *p*-Value
	Infected n = 87	Non-infected n = 122	
**Children**			
**Sex**			
Female	47 (54.0)	67 (54.9)	Reference
Male	40 (46.0)	55 (45.1)	1.04 (0.57–1.87), 1.000
**Diagnosis Age (year)**			
Median (IQR)	0.96 (0.12–2.58)	0.12 (0.10–0.19)	0.0001 *
**Birth weight (grams)**			
Median (IQR)	3190 (2783–3600)	2985 (2782–3283)	0.061
**Birth Weight Ranges**			
Normal (3000–3999)	55 (63.2)	59 (48.4)	Reference
Insufficient (2500–2999)	12 (13.8)	44 (36.1)	0.29 (0.13–0.64), 0.001 *
Low (1500–2499)	10 (11.5)	15 (12.3)	0.72 (0.26–1.87), 0.512
Overweight (>4000)	5 (5.7)	2 (1.6)	2.66 (0.41–29.04), 0.272
Very low (1000–1499)	1 (1.2)	0 (0.0)	Nc
Extremely low (<1000)	1 (1.2)	0 (0.0)	Nc
Ignored	3 (3.4)	2 (1.6)	Nc
**Delivery**			
Cesarean	36 (41.4)	87 (71.3)	Reference
Vaginal	48 (55.2)	35 (28.7)	3.29 (1.77–6.20), 5.1e^−05^
Ignored	3 (3.4)	0 (0.0)	Nc
**Breastfeeding**			
No	38 (43.7)	118 (96.7)	Reference
Yes	47 (54.0)	4 (3.3)	35.77 (11.96–145.38), 2.2e^−16^
Ignored	2 (2.3)	0 (0.0)	Nc
**Mothers**	**Transmitters** **N = 87**	**Non-transmitters** **N = 122**	
**Age at Delivery (years)**			
Median (IQR)	23.3 (20.0–29.4)	26.1 (22.9–31.4)	0.011*
**Diagnosis**			
Prenatal	19 (21.8)	56 (45.9)	Reference
Before prenatal	4 (4.6)	51 (41.8)	0.23 (0.05–0.77), 0.009 *
Delivery	19 (21.8)	14 (11.5)	3.94 (1.54–10.39), 0.002 *
Postpartum	45 (51.7)	1 (0.8)	126.55 (18.99–5257.76), 2.2e^−16^
**ART in Gestation**			
Yes	9 (10.3)	93 (76.2)	Reference
No	78 (89.6)	29 (23.8)	27.20 (11.81–69.62), 2.2e^−16^
**ART in Delivery**			
Yes	19 (21.8)	85 (69.7)	Reference
No	60 (69.0)	31 (25.4)	8.54 (4.27–17.75), 8.7e^−12^
Ignored	8 (9.2)	6 (4.9)	Nc
**Viral load in third trimester of pregnancy**			
Undetectable	1 (1.2)	35 (28.7)	Reference
Detectable	84 (96.5)	81 (66.4)	32.64 (5.17–1353.74), 7.4e^−8^
Ignored	2 (2.3)	6 (4.9)	Nc

OR = odds ratio; 95%CI = 95% confidence interval; n = sample number; * = significant *p*-value; IQR = interquartile.

**Table 2:** Allelic and genotypic distribution of *CCR5* (rs333) and *IFITM3* (rs12252) in children exposed to HIV-1 through vertical transmission and their respective mothers from northeast Brazil (Pernambuco state).

Genes/ Models	Children Exposed to HIV-1		Fisher’s Exact Test OR (95%CI), *p*-Value	Mothers		Fisher’s Exact Test OR (95%CI), *p*-Value
	Infected	Non-Infected		Transmitter	Non-Transmitter	
*CCR5∆32 (rs333)*	**n = 85**	**n = 103**		**n = 78**	**n = 112**	
*Allelic*						
*Wt*	166 (97.6)	203 (98.5)	Reference	153 (98.1)	28 (95.2)	Reference
*∆32*	4 (2.4)	3 (1.5)	1.63 (0.27–11.27), 0.706	3 (1.9)	6 (4.8)	0.71 (0.11–3.40), 0.742
*Codominant*						
*wt/wt*	81 (95.3)	100 (97.1)	Reference	75 (96.1)	106 (94.6)	Reference
*wt/∆32*	4 (4.7)	3 (2.9)	1.64 (0.27–11.53), 0.703	3 (3.9)	6 (5.4)	0.71 (0.11–3.44), 0.739
						
*IFITM3 (rs12252)*	*n* = *84*	*n* = *109*		*n* = *81*	*n* = *113*	
*Allelic*						
*T*	134 (79.8)	194 (89.0)	Reference	135 (83.3)	191 (84.5)	Reference
*C*	34 (20.2)	24 (11.0)	2.05 (1.12–3.79), 0.014 *	27 (16.7)	35 (15.5)	1.09 (0.60–1.95), 0.780
*Codominant*						
*TT*	54 (64.3)	86 (78.9)	Reference	55 (67.9)	79 (69.9)	Reference
*TC*	26 (30.9)	22 (20.2)	1.88 (0.92–3.86), 0.065	25 (30.9)	33 (29.2)	1.09 (0.55–2.12), 0.874
*CC*	4 (4.8)	1 (0.9)	6.29 (0.60–31.7), 0.080	1 (1.2)	1 (0.9)	1.43 (0.02–114.0), 1.000
*Dominant (TT vs TC+CC)*			2.07 (1.04–4.16), 0.034 *			1.10 (0.56–2.12), 0.875
*Recessive (TT+TC vs CC)*			5.36 (0.52–268.0), 0.169			1.40 (0.02–111.0), 1.000
*Over-dominant (TT+CC vs TC)*			1.77 (0.87–3.61), 0.095			1.08 (0.55–2.11), 0.874

OR = odds ratio; CI95% = 95% confidence interval; n = sample number; * = significant *p*-value; IQR = interquartile.

**Table 3:** Multivariable logistic regression results.

Variable	Odds Ratio	95% Confidence Interval		*p*-Value
		Lower Bound	Upper Bound	
**Model Intercept**	-	-	-	0.99
**Birth Weight**				
Normal	Reference			
Extremely low	1,101,362,729.76	0.00	Inf	1.00
Low	0.79	0.13	4.91	0.80
Insufficient	0.45	0.11	1.82	0.26
High	0.45	0.04	4.86	0.51
**Childbirth type**				
Vaginal	Reference			
Cesarean section	1.08	0.30	3.94	0.90
**Breastfeeding**				
No breastfeeding	Reference			
Breastfeeding	27.52	1.63	465.69	0.02
**Child *CCR5* genotypes**				
wt/wt	Reference			
wt/∆32	12,661,260.38	0.00	Inf	1.00
**Mother *CCR5* genotypes**				
wt/wt	Reference			
wt/∆32	0.94	0.05	16.38	0.96
**Child *IFITM-3* genotypes**				
TT	Reference			
CC	0.61	0.00	239.48	0.87
TC	4.25	0.48	37.40	0.19
**Mother *IFITM-3* genotypes**				
TT	Reference			
CC	0.00	0.00	Inf	1.00
TC	0.14	0.01	2.23	0.16
**Viral load at the last trimester of gestation**				
Undetectable	Reference			
Detectable	55,984,785.37	0.00	Inf	0.99
**Mother received HAART during childbirth**				
Yes	Reference			
No	1.80	0.50	6.48	0.37
**HIV-1 diagnosis**				
During prenatal care	Reference			
Before prenatal care	0.32	0.05	1.92	0.21
During childbirth	4.46	1.14	17.41	0.03
After childbirth	11.54	0.71	187.18	0.09
Interaction between mother and child *CCR5* genotypes	0.00	0.00	Inf	1.00
**Interaction between mother and child *IFITM3* genotypes**				
CC and CC	62,219,916,012,559,000.00	0.00	Inf	1.00
CC and TC	nc	nc	nc	nc
TC and CC	nc	nc	nc	nc
TC and TC	2.13	0.05	88.14	0.69

nc = not calculable.

## Data Availability

The data that support the findings of this study are available from the corresponding author upon reasonable request.

## Supplementary Materials

The following supporting information can be downloaded at: https://www.mdpi.com/article/10.3390/life13020397/s1, Table S1: CCR5 and IFITM3 genotypic concordance and discordance in children exposed to HIV-1 and their respective mothers.
