# Supplementary material for: Clinical–Epidemiological Characteristics and IFITM-3 (rs12252) Variant Involvement in HIV-1 Mother-to-Children Transmission Susceptibility in a Brazilian Population
Source: Life (Basel). 2023 Jan 31;13(2):397. doi: 10.3390/life13020397 (PMC9959554; doi:10.3390/life13020397)
Supplement: Supplementary file 1 [file life-13-00397-s001.zip › life-2141561-supplementary.pdf]

Supplementary Table S1. *CCR5* and *IFITM3* genotypic concordance and discordance in children exposed to HIV-1 and their respective mothers.

**Genotypes Children | mothers**	**Children Exposed to HIV-1**		**Fisher' Exact Test OR (95%CI), p-value**
	**Infected + transmitter**	**Non-infected + Non-transmitter**	
CCR5Δ32	***n=80***	***n=104***	
wt/wt|wt/wt	74	99	Reference
wt/Δ32|wt/Δ32	1	3	0.45 (0.01-5.70), 0.638
wt/wt|wt/Δ32	2	2	1.34 (0.09-18.82), 1.000
wt/Δ32| wt/wt	3	0	Nc
Concordant	75	102	Reference
Discordant	5	2	3.38 (0.53-36.35), 0.242
IFITM3	***n=78***	***n=101***	
TT|TT	46	63	Reference
TT|TC	6	17	0.49 (0.14-1.42), 0.168
TC|TC	17	12	1.93 (0.78-4.90), 0.143
TC|TT	6	7	1.17 (0.30-4.38), 0.777
CC|TC	2	1	2.71 (0.14-163.95), 0.575
CC|CC	1	0	Nc
TC|CC	0	1	Nc
Concordant	64	75	Reference
Discordant	14	26	0.63 (0.28-1.38), 0.278

OR = Odds ratio; CI95% = 95% Confidence Interval; n = sample number
